# TLR4 and prostaglandin pathways at the crossroads of endotoxemia-induced lipolysis

**DOI:** 10.3389/fimmu.2025.1591210

**Published:** 2025-05-19

**Authors:** Miguel Chirivi, Ursula Abou-Rjeileh, Madison Myers, Jair Parales-Giron, Lynn Worden, Adam L. Lock, G. Andres Contreras

**Affiliations:** ^1^ Department of Large Animal Clinical Sciences, College of Veterinary Medicine, Michigan State University, East Lansing, MI, United States; ^2^ Department of Animal Science, College of Agriculture and Natural Resources, Michigan State University, East Lansing, MI, United States

**Keywords:** lipolysis, prostaglandins, adipocytes, COX, endotoxemia, adipose tissue

## Abstract

**Background:**

During endotoxemia, immune activation rapidly increases energy needs. To meet these demands, white adipose tissue (WAT) mobilizes fatty acids through lipolysis. While fatty acids serve as energy-dense substrates, they also act as precursors for lipid mediators, including prostaglandins (PGs), that drive inflammation. The dual role of WAT is crucial during endotoxemia, wherein both energy needs and inflammatory signals are amplified. However, the mechanisms by which WAT regulates lipolysis during endotoxemia are not well understood. Dairy cows serve as an excellent model for studying endotoxemia due to the high incidence of the condition and increased susceptibility to lipolysis dysregulation observed during the periparturient period.

**Methods:**

Our study aimed to define the effects of endotoxemia on lipid mobilization and the regulatory role of PG receptors on the activity of key lipases within WAT. We used an *in vivo* lipopolysaccharide (LPS) infusion model of endotoxemia in lactating dairy cows (n = 4) to evaluate WAT inflammation, lipase activity, and PG receptor abundance 24 hours post-infusion. Additionally, we employed *in vitro* models using bovine adipocyte progenitor cells and mature adipocytes (n = 6) to investigate the effects of LPS exposure on lipid accumulation, lipolysis, arachidonic acid release, cyclooxygenase-2 (COX-2) activity, and EP receptor expression.

**Results:**

In lactating dairy cows, we observed enhanced WAT inflammation, reduced lipolysis, and decreased activity of adipose triglyceride lipase (ATGL) and hormone-sensitive lipase (HSL) 24 hours post-infusion. Notably, endotoxemia reduced the abundance of PG receptors EP2 and EP4 in WAT. Using the *in vitro* model, we found that adipocyte progenitor cells exposed to LPS during differentiation exhibited increased lipid accumulation after four days of adipogenic induction. In contrast, in mature adipocytes, LPS exposure (7 h) intensified lipolysis, an effect that was attenuated when toll-like receptor 4 (TLR4) was silenced. LPS also enhanced the release of arachidonic acid and adipocytes’ cyclooxygenase-2 (COX-2) activity, leading to increased biosynthesis of prostaglandin E2 (PGE_2_). LPS also increased the expression of EP2, a PGE_2_ receptor, while simultaneously reducing EP4 content. PGE_2_ activated lipolysis in an EP4 receptor-dependent manner. COX inhibition reduced the biosynthesis of PGE_2_, inhibited lipolysis, and upregulated EP4 expression.

**Conclusion:**

These data demonstrate that, during endotoxemia, TLR4 activation in bovine adipocytes triggers lipolysis via prostaglandin E2-dependent mechanisms. In addition, LPS modulates EP receptor expression, resulting in alterations in lipid mobilization. Together, these data provide initial evidence of prostaglandin receptors as possible targets for modulating WAT lipid trafficking during endotoxemia.

## Graphical Abstract

Effects of lipopolysaccharide (LPS) exposure on white adipose tissue (WAT) lipid metabolism during endotoxemia in a dairy cow model. The timeline highlights the biphasic response: Upon LPS stimulation, toll-like receptor 4 (TLR4) activation triggers cyclooxygenase-2 (COX-2) activity, leading to increased prostaglandin E2 (PGE2) synthesis. In the short term, PGE2 promotes lipolysis via EP4 receptor activation. Over time, LPS exposure downregulates EP4, resulting in reduced lipolysis and increased lipid accumulation in adipocytes. These findings suggest that targeting PGE2 signaling may help modulate lipid mobilization during endotoxemia.

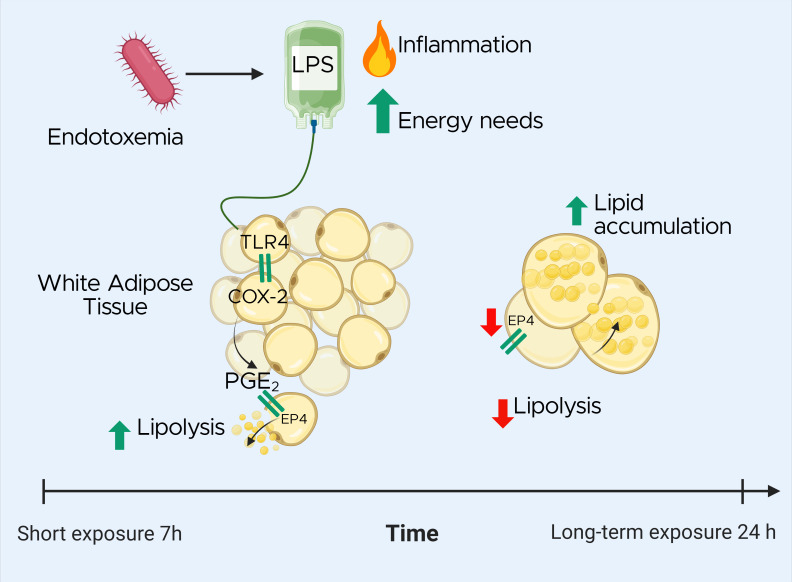

## 1 Introduction

Endotoxemia is defined as the elevation of plasma levels of endotoxins that produce a systemic inflammatory response. Lipopolysaccharide (LPS), a key component of the outer membrane of Gram-negative bacteria, is the most common and well-studied endotoxin. Endotoxemia affects 5–7 million human patients annually, with mortality rates ranging from 50 to 70% (1). In companion animals and livestock, the prevalence of endotoxemia is not well-documented and, therefore, challenging to estimate. However, in severe cases, mortality rates range from 20 to 68%, depending on the species and associated conditions (2–4). In addition to inducing acute inflammatory responses, endotoxemia is strongly associated with metabolic disorders, including insulin resistance, liver dysfunction, ketosis (as seen in dairy cows), and obesity in humans. This association is attributed, in part, to significant disturbances in white adipose tissue (WAT) function (5). Farm animals such as dairy cows are particularly prone to developing endotoxemia around parturition due to various risk factors, including ruminal and intestinal bacterial translocation and the high incidence of mastitis, metritis, and heat stress associated with elevated plasma LPS levels (6, 7). Cows, like humans, undergo significant WAT alterations during endotoxemia, including disturbances in fatty acid mobilization and increased macrophage infiltration that, together, increase the risk for metabolic disorders (6, 8).

In mammals, WAT regulates energy storage and release to meet bioenergetic needs. During endotoxemia and other systemic inflammatory conditions, WAT lipolysis serves a dual purpose: it mobilizes fatty acids to supply energy for immune activation (9), and releases substrates for the synthesis of lipid mediators, such as prostaglandins, leukotrienes, and other oxylipin species, that modulate the inflammatory response (10). Therefore, lipolysis may be especially important in the context of endotoxemia, wherein both energy demands and inflammatory signals are amplified. Although excessive lipolysis is known to exacerbate WAT inflammation across species, the complex interplay between endotoxemia and the mechanisms that govern lipolysis and the biosynthesis of inflammatory lipid mediators remain largely uncharacterized (11, 12).

Lipolysis encompasses the hydrolysis of triglycerides (TG) through the activation of two rate-limiting neutral lipases: adipose triglyceride lipase (ATGL), the rate-limiting enzyme in basal lipolysis; hormone-sensitive lipase (HSL), the rate-limiting enzyme during demand-driven lipolysis (13–15). The canonical pathway of lipolysis is mainly activated by catecholamines and natriuretic peptides. Once catecholamines bind to β-adrenergic receptors (β-ARs) within the membrane of adipocytes, intracellular levels of cyclic AMP (cAMP) surge due to adenyl cyclase activation. The accumulation of cAMP triggers the stimulation of protein kinase A (PKA), which phosphorylates HSL and perilipin-1 (PLIN-1). Importantly, the phosphorylation of PLIN-1 releases the coactivator α-β hydrolase domain containing 5 (ABHD5) from PLIN-1, allowing for the activation of ATGL (16). However, PKA is not the only kinase capable of phosphorylating HSL. During inflammatory conditions, cytokines and pathogens including endotoxins activate ERK1/2, resulting in phosphorylation of HSL and stimulation of lipolysis (17, 18). ATGL is recognized as a key enzyme for energy homeostasis throughout the body with multiple physiological functions in lipid and energy metabolism. However, ATGL’s role extends well beyond energy metabolism; it also regulates the inflammatory response in macrophages and mast cells by modulating the production of arachidonic acid (Ara) -derived oxylipins (i.e., eicosanoids (19, 20);. Despite these findings, it is unknown whether the activation of ATGL is regulated to minimize lipotoxicity and limit inflammation in WAT during endotoxemia.

Polyunsaturated fatty acids (PUFA) released during lipolysis can be metabolized by oxidative enzymes including cyclooxygenases (COX), lipoxygenases (LOX), and cytochrome P450s (CYP450), into bioactive lipid mediators known as oxylipins (21). These compounds are known to modulate WAT function during inflammatory conditions, but their immunomodulatory effects extend far beyond WAT. Among the oxylipins, prostanoids originate from arachidonic acid (C20:4, n-6) and are produced through the action of COX and prostanoid synthases (22). At present, two COX isoforms have been identified: COX-1 and COX-2. While COX-1 is ubiquitously expressed, COX-2 expression and activity are triggered by inflammatory cytokines such as IL-1β and TNF-α, in addition to inflammatory molecules including LPS. COX activity results in the synthesis of four types of prostaglandins (PG), PGD_2_, PGE_2_, PGF_2_, and PGI_2_, along with thromboxanes. Among these, PGE_2_ is the most abundantly PG synthesized during lipolysis (21). PGE_2_ exerts its effects by binding to four subtypes of G protein-coupled rhodopsin-type E prostanoid receptors (EP): EP1, EP2, EP3, and EP4. The biological actions of EP within WAT depend on the type of receptor activated (23). For instance, EP1 is coupled to G_q_ proteins which activate phospholipase C, stimulating the mobilization of calcium; whereas EP3, which is coupled to G_i_ proteins, inhibits adenylate cyclase, reducing cAMP levels and, thereby limiting lipolysis (24). In contrast, EP2 and EP4 are coupled to G_s_ proteins which activate adenylyl cyclase, with the latter playing a role in lipolysis activation (25). While the role of COX-2 in inflammation is well established, a lack of understanding regarding the function of COX-2 in lipid mobilization in the context of endotoxemia remains. We hypothesize that endotoxin-induced inflammation modulates the production of PG and the expression and activity of EP, which, in turn, regulates lipolysis.

In the present study, we determined that endotoxin enhances the production of prostanoids and impairs lipolytic activity in WAT using *in vivo* and *in vitro* bovine models of endotoxemia. In addition, we provide initial evidence that LPS-induced lipolysis is mediated by COX activity in adipocytes. Furthemore, exposure to LPS alters the expression of EP receptors in WAT, which may serve to attenuate lipolysis during intense inflammation. Our findings provide novel insights into the complex regulatory networks that govern lipid metabolism during LPS-triggered inflammation and highlight the potential of PG and EP as therapeutic targets for mitigating the metabolic dysregulation associated with endotoxemia.

## 2 Materials and methods

### 2.1 Bovine endotoxemia model

#### 2.1.1 Animals

All experimental procedures were approved by the Institutional Animal Care and Use Committee (IACUC #201900248) at Michigan State University, (East Lansing, MI). We enrolled eight multiparous Holstein dairy cows from the Michigan State University Dairy Cattle Teaching and Research Center for this study. The averages (mean ± SD) in days lactating were 204 ± 21, body condition score (BCS) of 3.26 ± 0.15, previous lactation 305-days mature-equivalent yield (MEq) of 12,438 ± 910 kg, and parity 2.5 ± 1.06. BCS were recorded by three trained investigators using a 5-point scale, as described by Wildman et al. (26). Previous lactation 305-days MEq refers to the total amount of milk a cow produced during her last lactation period, standardized to a typical 305-d lactation. Animals showing signs of systemic infection, gastrointestinal diseases, respiratory conditions, any clinical signs of illness, and BCS higher than 3.6 and lower than 2.9. were excluded from the study. Throughout the experiment, cows were housed in individual tie-stalls bedded with sawdust (cleaned twice daily), milked three times daily (at 0400, 1200 and 2000 h), and had water available ad libitum. All animals received a common lactation diet formulated to meet their energy requirements (27). At 0700 h daily, cows were fed at 115% of their expected intake. Feed access was blocked once per day for collection and measurement of orts (uneaten feed). Standard health herd checks were maintained during the study.

#### 2.1.2 *In vivo* model of endotoxemia

The animal portion of the present study included two experimental periods (P1, P2). During P1 (5 d), cows were acclimated, baseline production data were collected, and a jugular catheter was inserted as described previously (28). At the beginning of P2 (7 d), cows received intravenous (IV) infusions of either 100 mL of saline solution (SS: sterile sodium chloride solution 0.9%; n = 4) or lipopolysaccharide (LPS: 1 µg LPS/kg BW*; E. coli* O55:B5; Cat N° L6529, Sigma-Aldrich, St. Louis, MO; n = 4) dissolved in 100 mL of SS. Treatments were administered continuously for over 20 min. The LPS dose and bacterial serotype were selected based on prior research (18).

#### 2.1.3 Sample and data collection

Blood was collected via coccygeal venipuncture using coated collection tubes (K2 EDTA) prior to morning feeding. Blood samples were collected once daily during P1 and at 0, 2, 4, 6, 12, 24, 48, 72, 96 and 120 h relative to IV infusion during P2. Samples were centrifuged at 2000 × *g* for 10 min at 4°C for plasma fraction collection and then stored at −80°C until further analysis. Subcutaneous WAT samples (~ 5 g) were collected 24 h after IV infusion from the right flank following our established protocol (18). As described below, WAT samples were used for ex vivo lipolysis assay, protein, transcriptional, and histological analyses. Vital physiological parameters, including rectal temperature, respiration rate, and heart rate were recorded once per day in P1 and hourly for the first 6 h post-infusion, then at 12 h, and subsequently every 24 h in P2. Rectal temperatures were measured using a digital thermometer. Respiratory rates were determined by counting flank movements for a 30-s interval and multiplying that count by 2 to convert to respirations per min. Heart rates were determined by using a stethoscope placed on the left side of the thorax near the heart girth region. Heart beats were counted for a 30-s interval and multiplied by 2 to convert to beats per min. Milk yield and orts were recorded daily throughout the experiment. Body weights (BW) were recorded three times per week. Plasma concentration of LPS binding protein (LBP), serum amyloid A (SAA), haptoglobin (Hp), non-esterified fatty acids (NEFA), β-hydroxybutyrate (BHB), glucose, calcium, and magnesium were quantified as described previously by our group (7).

#### 2.1.4 Ex vivo lipolysis assay

After collection, WAT was transported to the laboratory at 37°C in Krebs Ringer Bicarbonate HEPES Buffer (KRBH, pH 7.4) to assess AT lipolysis as detailed previously (18). Briefly, WAT was cut into approximately 100 mg fragments which were individually placed in 24-well plate wells containing 1 mL of KRBH supplemented with 3% fatty acid-free bovine serum albumin (BSA, Millipore-Sigma, Burlington, MA). Basal lipolysis was determined without any additional reagents, serving as experimental control (CON). To assess stimulated canonical lipolysis, WAT fragments were challenged with 1 µ*M* isoproterenol hydrochloride (ISO; Cat N° I6504, Millipore-Sigma, Burlington, MA), a β-adrenergic agonist. Insulin sensitivity of WAT was evaluated by treating explants with 1 µg/L bovine insulin (INS; Cat N° I0516, Sigma-Aldrich) 1 h prior to and during ISO stimulation. Following 3 h of ISO stimulation, culture media was collected, snap-frozen in LN_2_, and stored at −80°C until further analysis.

#### 2.1.5 Immunohistochemistry

WAT samples were immediately fixed in 4% paraformaldehyde, embedded in paraffin, and sectioned into 4 µm slices at the Michigan State University Investigative Histopathology Laboratory. To assess macrophage infiltration, WAT sections were incubated overnight at 4°C with a mouse monoclonal antibody against bovine CD172a (1:50; Cat N° DH59B, Washington State University Monoclonal Antibody Center, Pullman, WA) diluted in normal antibody diluent (Scytek). Sections were counterstained with CAT Hematoxylin (1:10; Cat N° 50-823-94, Biocare, Concord, CA). Digital images of whole slides were captured using the Olympus VS200 Research Slide Scanner (Olympus Americas, Center Valley, PA). The area of individual adipocytes was measured in 8 random fields (1.125 mm² each) per section using the Adiposoft plugin in ImageJ, with sizes categorized into 7 bins. CD172a intensity was also quantified using ImageJ. All analyses were conducted by trained technicians blinded to experimental treatments, as previously described by our group (29).

### 2.2 *In vitro* model

#### 2.2.1 Isolation of adipocyte progenitors

Nine healthy, multiparous, non-gestating, non-lactating Holstein dairy cows from commercial herds in Michigan were selected from a local abattoir. All animals had a BCS between 3.25 to 3.75. Following euthanasia by captive bolt and jugular exsanguination, internal organs of candidate animals were evaluated. Animals with evidence of intra-abdominal, thoracic, or gastrointestinal disease were excluded. Following hide removal, approximately 50 g WAT explants were harvested from the right paralumbar fossa (flank) region. Isolation of adipocyte progenitor (AP) cells was performed through the outgrowth of explants as described in (30).

#### 2.2.2 TLR4 silencing

Isolated AP cells were then seeded in 6-well cell culture plates (Corning Costar Corp., Cambridge, MA) at 20,000 cells/cm^2^ and incubated overnight in alpha minimum essential medium (α-MEM; Cat N° 50-010-PB, Corning), supplemented with 5% FBS, HiPerFect transfection reagent (Cat N° 301704, Qiagen, Hilden, Germany), and 40 nM of three combined siRNA sequences targeting *TLR4* (si*TLR4*), or a non-coding siRNA (siNC). Sequences were designed by IDT Technologies (Coralville, IA; **Supplementary Table S1**). After incubation, the medium was replaced with preadipocyte media, and cells were allowed to proliferate for 48 h until confluency was reached (31).

#### 2.2.3 Adipogenesis

AP cells were seeded in 6-well cell culture plates at 20,000 cells/cm^2^, allowed to proliferate until confluent, and then induced to differentiate as reported previously (32). The AP cells were treated with 1 µg/mL of LPS from *Escherichia coli* O55:B5 (LPS; Cat N° L6529, Sigma-Aldrich) and adipogenesis was evaluated after 4 d using Bodipy 493/503 (Cat N° D3922, ThermoFisher, Waltham, MA), a neutral lipid stain, and the nuclear stain NucSpot^®^ Live 650 (Cat N° 40082, Biotium, San Francisco, CA). Adipogenic efficiency is reported as Bodipy fluorescence intensity/nuclei count using the long-term, live-cell imaging IncuCyte^®^ S3 System (Sartorius). Quantification of cell images was performed after 4 d using the IncuCyte ZOOM™ software.

#### 2.2.4 Lipolysis assays

1. To investigate LPS’s capacity to induce lipolysis and determine the optimal dose for use in subsequent experiments, mature adipocytes (AP differentiated for 7 d) were incubated for 3 h in lipolysis media [Krebs Ringer Bicarbonate-Buffered solution (KRBB) + 2% BSA] containing 0, 0.01, 0.1, 1 and 10 µg/mL of LPS. Based on these preliminary tests, a dose of 1 µg/mL was selected for further experiments as it effectively induced lipolysis.
2. To evaluate the role of TLR4 in stimulated lipolysis, 7-d si*TLR4* and siNC were incubated for 3 h in lipolysis media containing 1 µg/mL of LPS or the beta-adrenergic agonist isoproterenol at 1 µ*M* (ISO; Cat N° I6504, Millipore-Sigma). ISO was used as positive control for canonical lipolysis and basal lipolysis was established without the addition of any reagent (control=CON).
3. To investigate adipocyte’s insulin sensitivity during lipolysis, adipocytes differentiated for 7 d were incubated 60 min with 1 µg/L of insulin from bovine pancreas (INS; Cat N° I0516, Sigma-Aldrich). Next, adipocytes were incubated for 7 h in lipolysis media treated with LPS (INS+LPS), or ISO (INS+ISO).
4. To inhibit lipolysis and COX activity, 7-d-differentiated adipocytes were pre-treated during 2 h with 100 µ*M* of niacin (a GPR109A agonist; NIA; Cat N° HY-B0143, MedChemExpress, Monmouth Junction, NJ), or 10 µ*M* of the COX inhibitor flunixin meglumine (FM; Cat N° HY-B0386, MedChemExpress), or the combination NIA+FM. Then, adipocytes were treated with ISO and LPS as described above.
5. To assess the effects of PGE_2_ and its receptors on lipolysis activation, 7-d-differentiated adipocytes were exposed for 3 h to 100 m*M*, 500 m*M* and 1 µ*M* of PGE_2_ (PGE_2_; Cat N° 14010), and 10 m*M* of EP agonists 17-phenyl trinor prostaglandin E2 (EP1; Cat N° 14810), 11-deoxy prostaglandin E1 (EP2; Cat N° 13510), Sulprostone (EP3; Cat N° 14765), and CAY10684 (EP4; Cat N° 15966) from Cayman Chemical Ann Arbor.

After incubation times described above elapsed, cell culture media was collected, snap-frozen in LN_2_, and stored at -80 °C.

### 2.3 Lipolysis quantification

Lipolysis was assessed by quantification of glycerol released in both WAT explant and cell culture media, using Glycerol-Glo™ Assay (Cat N° J3151, Promega Corp., Madison, WI) following manufacturer recommendations. The intra- and inter-assay coefficients of variation were 4.71% and 12.63%, respectively. Glycerol was normalized by AT weight (µM/mg) in explants and by the content of protein in cultured cells, respectively. Results are expressed as a ratio relative to CON glycerol release.

### 2.4 RNA extraction

Whole tissues were homogenized in Trizol (Cat N° 15596026, Invitrogen Waltham, MA) using a bead mill tissue homogenizer (FisherScientific) as described previously (18). Following homogenization, chloroform was added and incubated prior to centrifugation at 12,000 × *g* for 15 min at 4°C. Total RNA extraction on the aqueous phase of the centrifuged homogenate was performed using Quick-RNA Miniprep plus kit (Cat N° R1058, Zymo Research) according to the manufacturer’s instructions.

For cells, RNA was extracted using the Maxwell^®^ RSC simplyRNA cells kit (Cat N° AS1390, Promega Corp.), following manufacturer protocol. To eliminate genomic DNA, 5 µL of DNase I (Promega) was added. Automated extraction of RNA was carried out using the Maxwell^®^ RSC instrument (Promega Corp.). RNA was flash-frozen in LN_2_, stored at -80°C, and the concentration and integrity of total RNA were evaluated using a NanoDrop One^©^ spectrophotometer (Cat N° 840274200; Thermofisher Scientific). All samples had a 260:280 nm ratio between 2.01 and 2.04 and RNA integrity value >9.

### 2.5 Bulk RNA sequencing

A subset of CON-, ISO-, and LPS-treated adipocyte samples was randomly selected from 3 animals for next-generation bulk RNA sequencing analysis (RNA-seq). The bioinformatic methods performed are described in detail in (30). All data are available in the NCBI Gene Expression Omnibus under accession number GSE267141.

### 2.6 qPCR

Reverse transcription was performed using 500 ng of sample RNA using 4 µL of the qScript cDNA SuperMix (Cat N° 95048, Quantabio, Beverly, MA) cycled for 5 min at 25°C, 30 min at 42°C, and 5 min at 85°C. Sample cDNA was stored at -20°C. Transcriptional studies were performed as previously described (18). Samples were assayed in duplicate using the QuantStudio 3 Real-Time PCR System (Cat N° A28567, Applied Biosystems, Waltham, MA). Each 10 µL PCR reaction contained 1X (5 µL) of SYBR™ Green PCR Master Mix (Cat N° 4309155, Applied Biosystems), 400 n*M* of primer assays (**Supplementary Table S1**), and 8 ng of sample cDNA. Transcriptional studies for *IRS1, PTGER2, PTGER4* and *PTGS2* were performed using the ABI QuantStudio 7 Flex Real-Time PCR system. Each 10 µL of reaction contained 1X (5 µL) of Prime Time™ Gene Expression Master Mix (Cat N° 1055772, Integrated DNA Technologies, Coralville, IA), 1X TaqMan Prime Time qPCR Assay (**Supplementary Table S1**) and 8 ng of sample cDNA. A non-template control and non-reverse-transcriptase control were used to monitor contamination and primer-dimer formation that could produce false-positive results and validate the absence of genomic DNA. Reference genes with the lowest pairwise variation values including eukaryotic translation initiation factor 3 subunit K (*EIF3K*), ribosomal protein lateral stalk subunit P0 (*RPLP0)*, and ribosomal protein S9 (*RPS9*) were used. Expression CT values for all genes of interest were normalized against the geometric mean of CT values from the selected housekeeping genes. Quantification cycle values were calculated using the 2^-ΔΔCT^ method.

### 2.7 Protein analysis

Protein was extracted from ∼100 mg of snap-frozen WAT samples homogenized in RIPA buffer (Cat N° R3792, Teknova, Hollister, CA) containing Halt™ protease inhibitor (Thermo Fisher Scientific) as described previously (33). Protein from cultured adipocytes was extracted from one well/treatment per cow of a 6-well palate using RIPA buffer containing protease inhibitors. Estimation of protein content was carried out using the Pierce BCA Protein Assay Kit (Cat N° 78429, Thermo Fisher Scientific). The protein concentration was determined using the Pierce™ BCA protein assay kit (Cat N° 23225, Thermo Scientific). The optimal protein concentration for the antibodies used in these experiments was between 0.5 and 0.75 mg/mL as established in the 12–230 kDa Abby™ Separation Module capillary cartridges of the Protein Simple Abby™ system [Cat N° SM-W004, ProteinSimple, Santa Clara, CA (34)]. Antibodies and dilutions are described in **Supplementary Table S2**. Sample proteins were separated by microcapillary electrophoresis and the chemiluminescence signal peaks were generated for analysis as described before (33). Targeted proteins were estimated and normalized against the total protein detected with the total protein detection module kit (Cat N° DM-TP01, ProteinSimple). The normalized data are expressed as relative to CON value, and ratios pHSL(Ser563):HSL and pERK: ERK. Positive controls were over-expression lysates for HSL, and ERK obtained from OriGene Technologies (Rockvilly, MD) while COX-2, PTGER2, and PTGER4 were from Abnova Corporation (Taipei City, Taiwan).

### 2.8 Lipidomics

Plasma and cell culture media samples were collected, extracted, and analyzed using high performance liquid chromatography tandem mass spectrometry (HPLC-MS/MS) to quantify prostaglandin E2 (PGE_2_) and ArA abundance as described in detail in **Supplementary Methods**.

### 2.9 Statistical analysis

Plasma data were analyzed using a Mixed model in JMP (JMP^®^ v.17; SAS Institute, Cary, NC). Variables evaluated over time were analyzed with repeated measures and the random effect of cow, the fixed effect of infusion, time, and their interactions. Data from AT biopsies were analyzed with a non-parametric Mann-Whitney test to determine the statistical differences between LPS and Control cows. *In vitro* data were analyzed by one- or two-way ANOVA and Tukey’s *post-hoc* adjustment was used for pairwise comparisons. The normality of the variables was checked using the Shapiro-Wilk Test (p < 0.05). Residuals of the models were checked and found to be normally distributed. Residuals for the lipidomics analysis were not normally distributed. These variables were log_10_-transformed for statistical analysis, and model fit was reassessed. Results are presented as mean ± SEM unless stated otherwise. Significance was declared at p ≤ 0.05, and tendencies were declared at p ≤ 0.10.

RNAseq data was analyzed using the DESeq2 package (v.1.20.0) in R (R Foundation for Statistical Computing, Vienna, Austria), based on negative binomial distributions (35). DESeq2-normalized gene counts were log_2_-transformed, and Student’s t-tests were applied, with P-values adjusted using Benjamini and Hochberg’s false discovery rate (FDR) correction. Genes were identified as differentially expressed (DEG) if the log_2_-fold-change (FC) exceeded 0.58 (FC > 1.5) and the adjusted p-value was ≤ 0.05 [−log_10_(adjusted p-value > 1.30103)]. Gene Ontology (GO) enrichment was conducted via ShinyGO (v0.80 Software, South Dakota State University, Brookings SD) for functional clustering of DEGs, with significance set at p < 0.05. Kyoto Encyclopedia of Genes and Genomes (KEGG) pathway analysis was included, with results detailed in Additional File 2. Volcano plots were generated in GraphPad Prism (v.10 for Windows; GraphPad Software, Boston, MA) to illustrate differences in DEG across comparison groups. One-way ANOVA with Fisher’s LSD was performed using DESeq2-normalized counts as described before (36).

## 3 Results

### 3.1 *In vivo* model of endotoxemia

#### 3.1.1 Endotoxemia induces systemic and WAT inflammation

**Figure 1:**
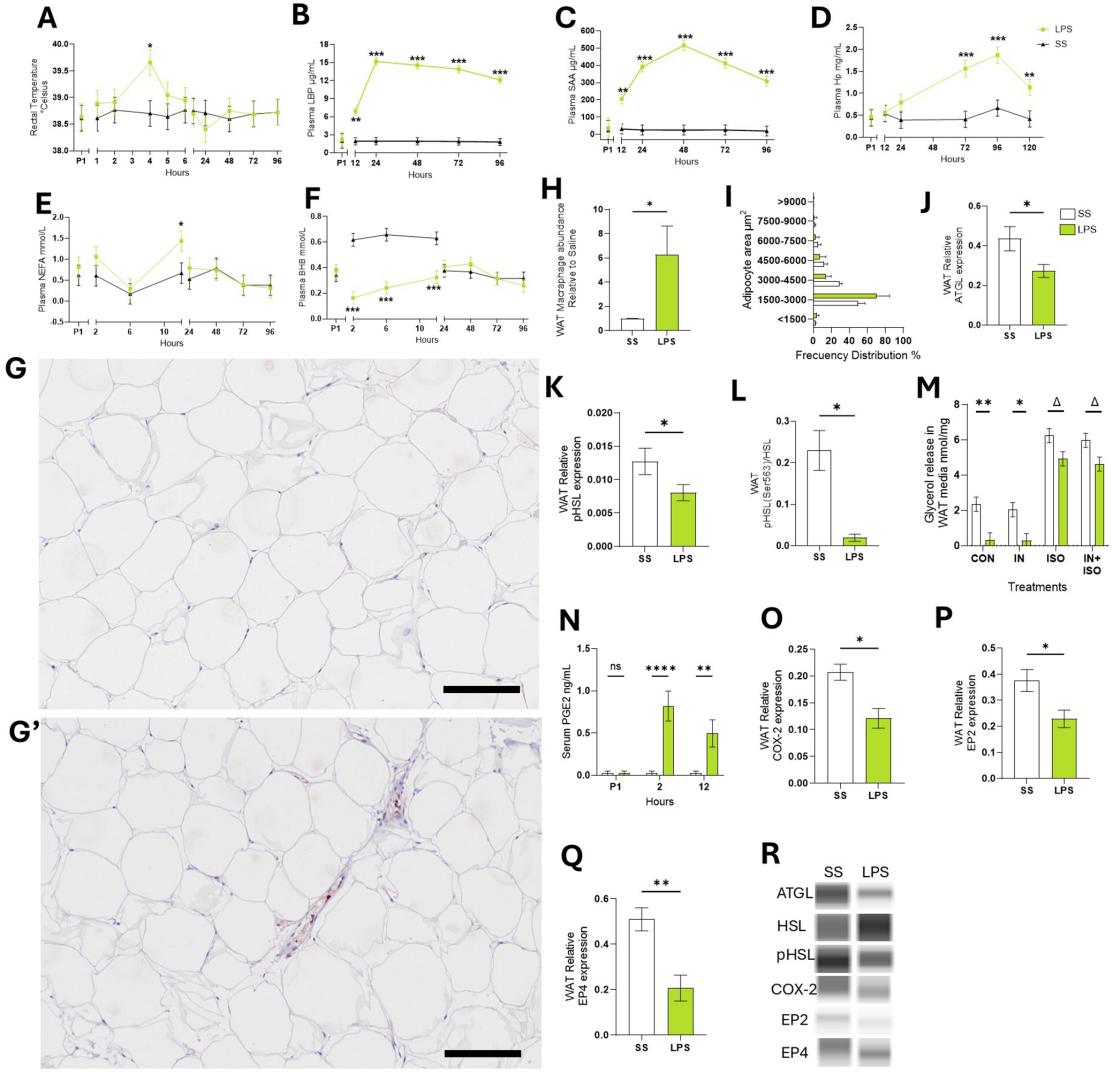
Endotoxemia induced systemic and sWAT inflammation. **(A)** Rectal temperature. **(B)** LPS binding protein – LBP. **(C)** Serum amyloid A – SAA. **(D)** Haptoglobin – Hp. **(E)** Non-esterified fatty acids – NEFA. **(F)** Beta-hydroxybutyrate – BHB. (G-G’) Representative images of WAT tissue sections after immunohistochemical staining using CD172a antibody to detect WAT macrophages in SS **(G)** or LPS cows (G’). **(H)** Abundance of CD172a antibody signal in WAT sections relative to SS. **(I)** Frequency distribution of adipocyte area calculated in WAT sections. **(J)** WAT protein abundance of adipose triglyceride lipase –ATGL. **(K)** WAT Protein abundance of phosphorylated hormone sensitive lipase – pHSL, **(L)** ratio protein pHSL: HSL. **(M)** Glycerol release by WAT during ex-vivo lipolysis assay. **(N)** Serum concentrations (ng/mL) of prostaglandin E2 – PGE2. **(O)** WAT Protein abundance of cyclooxygenase-2 – COX-2. **(P)** WAT Protein abundance of PGE2 receptor 2 – EP2. **(Q)** WAT Protein abundance of PGE2 receptor 4 – EP4. **(R)** Representative blots obtained in Abby protein Simple System. Data are represented as mean ± SEM. Bars with * differ *P* < 0.05, **P < 0.01, or ***(*P* < 0.001), or Δ (*P* < 0.1) n = 4. Time P1 represents the average of measurements obtained during period 1 and was not used as a covariate Scale bars: 100 μm (G, G’). Protein was normalized with the total protein content quantified with the Abby protein Simple System.

Compared to SS, LPS infusion increased rectal temperature by 0.9°C in cows compared to SS during the first 4 hours (p < 0.05; **Figure 1A**). LPS increased respiration rates by 70% within the first 2 h after infusion (p < 0.001; **Supplementary Figure S1A**). Compared to SS, LPS increased plasma concentrations of the acute phase proteins LBP, SAA, and Hp. The greatest changes occurred at 24 hours for LBP (8-fold increase, p < 0.001), at 48 hours for SAA (21-fold increase, p < 0.001), and at 96 hours for Hp (4-fold increase, p < 0.001; **Figures 1B-D**). LPS infusion reduced appetite (i.e., dry matter intake) by 26.2% (p < 0.001) and milk production by 12.9 ± 1.3 kg per d for 5 d following infusion (p < 0.001; **Supplementary Figures S1B, C**). LPS did not alter BW or BCS up to 6 d after infusion (data not shown).

After 12 h of IV treatment, LPS raised plasma NEFA by 73.2% compared to SS (p < 0.05; **Figure 1E**). As expected during the first 12 h, LPS reduced BHB (p < 0.001; -61.9%; **Figure 1F**), glucose (-14.8%; p <0.01), and calcium (-27.8%; p < 0.001) and lowered Mg by 40.9% at 96 h post-infusion (p < 0.001; **Supplementary Figures S1D-F**) relative to SS.

Macrophage infiltration was assessed in WAT sections by immunohistochemistry using an antibody against the macrophage marker CD172a, labeled with HRP. Compared to SS, LPS increased HRP signal 5.4-fold change (p < 0.05; **Figures 1G, H**). Adipocyte size distribution, which serves as an indicator of lipolysis and triglyceride storage within fat cells, was not different between LPS and SS (p > 0.1; **Figure 1I**).

#### 3.1.2 Endotoxemia modulates neutral lipase activity in WAT

In WAT collected 24 h post-infusion, LPS reduced ATGL activity—the rate-limiting enzyme in basal lipolysis—by 33.5% (p < 0.05; **Figure 1J**). LPS also decreased the ratio of phosphorylated HSL: total HSL (pHSL: HSL) by 36.7% (p < 0.05; **Figures 1K, L**). Ex-vivo, WAT from LPS treated cows exhibited 85.9% lower basal lipolysis than WAT collected from SS cows (p < 0.01) and tended to reduce ISO-stimulated lipolysis intensity by 21.03% (p = 0.07; **Figure 1M**). INS did not reduce ISO-stimulated lipolysis in WAT from LPS or SS cows (p > 0.1).

#### 3.1.3 Endotoxemia alters prostaglandin E2 pathway in WAT

Compared to SS, LPS increased blood concentrations of ArA-derived products generated through COX activity including PGE_2_ (p < 0.01; **Figure 1N**). LPS decreased COX-2 protein expression in WAT by 40% compared to SS cows (p < 0.05; **Figure 1O**). Additionally, LPS reduced the expression of PGE_2_ receptors 2 (EP2) and 4 (EP4) by 38.2% and 59.6%, respectively (p < 0.05; **Figures 1P, Q, R**).

### 3.2 Endotoxin alters adipocyte’s metabolic function

#### 3.2.1 Effects on transcriptomic profile

**Figure 2:**
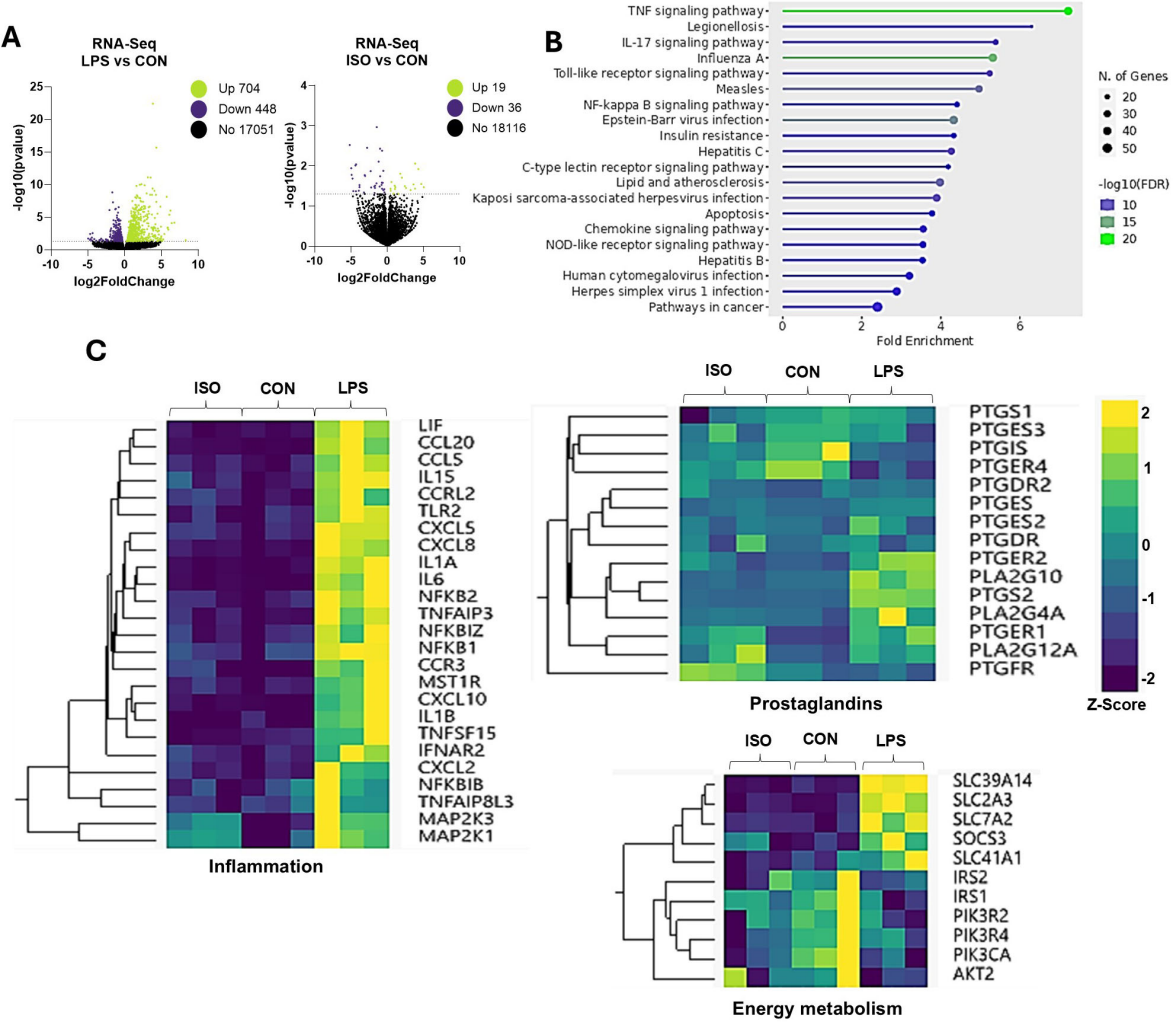
Effects of LPS on Transcriptomic Profile in Bovine Adipocytes. Differentiated (7-d) bovine adipocytes (n = 6) were treated with media control (CON), Lipopolysaccharide (LPS = 1 µg/mL) or isoproterenol (ISO = 1 µM) for 7 h. **(A)** Volcano plots of differentially expressed genes with LPS and ISO when compared to CON adipocytes. Each dot represents a gene **(B)** Enrichment of differentially expressed genes (DEG) using ShinyGO 0.77 Software in adipocytes treated with LPS vs CON **(C)** Selected gene networks clusters for inflammation, synthesis and receptors of prostaglandins and energy metabolism. Values are Z-scores (standards scores) of genes counts.

Next, we evaluated the direct effect of LPS on adipocytes’ gene expression *in vitro*. LPS exposure altered the transcription pattern of 1,152 genes, with 704 genes upregulated, and 448 downregulated (*p* < 0.05; **Figure 2A**). In response to canonical lipolysis stimulation with ISO, 19 genes were upregulated and 36 were downregulated. Functional enrichment analysis revealed that LPS induced differential expression of key genes grouped into functional categories including TNF, toll-like receptor, insulin resistance, and nuclear factor kappa B signaling pathway (p < 0.05; **Figure 2B**). LPS upregulated the transcription of inflammatory genes related to the innate immune response, chemoattractant factors, interleukins, and components of the mitogen-activated protein kinase cascade (**Figure 2C**).

Exposure to LPS also enhanced the transcription of the A2 family of phospholipases, including *PLA2G4A* and *PLA2G10.* Similarly, LPS upregulated the expression of *PTGS2*, encoding for the prostaglandin-endoperoxide synthase 2 (cyclooxygenase-2 or COX-2). Interestingly, LPS upregulated *PTGER2* (EP2) and downregulated *PTGER4* (EP4; **Figure 2C**). Further, LPS downregulated insulin receptor substrate (IRS) genes *AKT2* and *PIK3CA.* On the other hand, LPS upregulated the transcription of several solute carrier (SLC) transporter genes, including *SLC39A14, SLC2A3, SLC7A2*, and *SLC41A4* (*p* < 0.01; **Figure 2C**).

#### 3.2.2 Effects on adipogenesis and lipolysis

**Figure 3:**
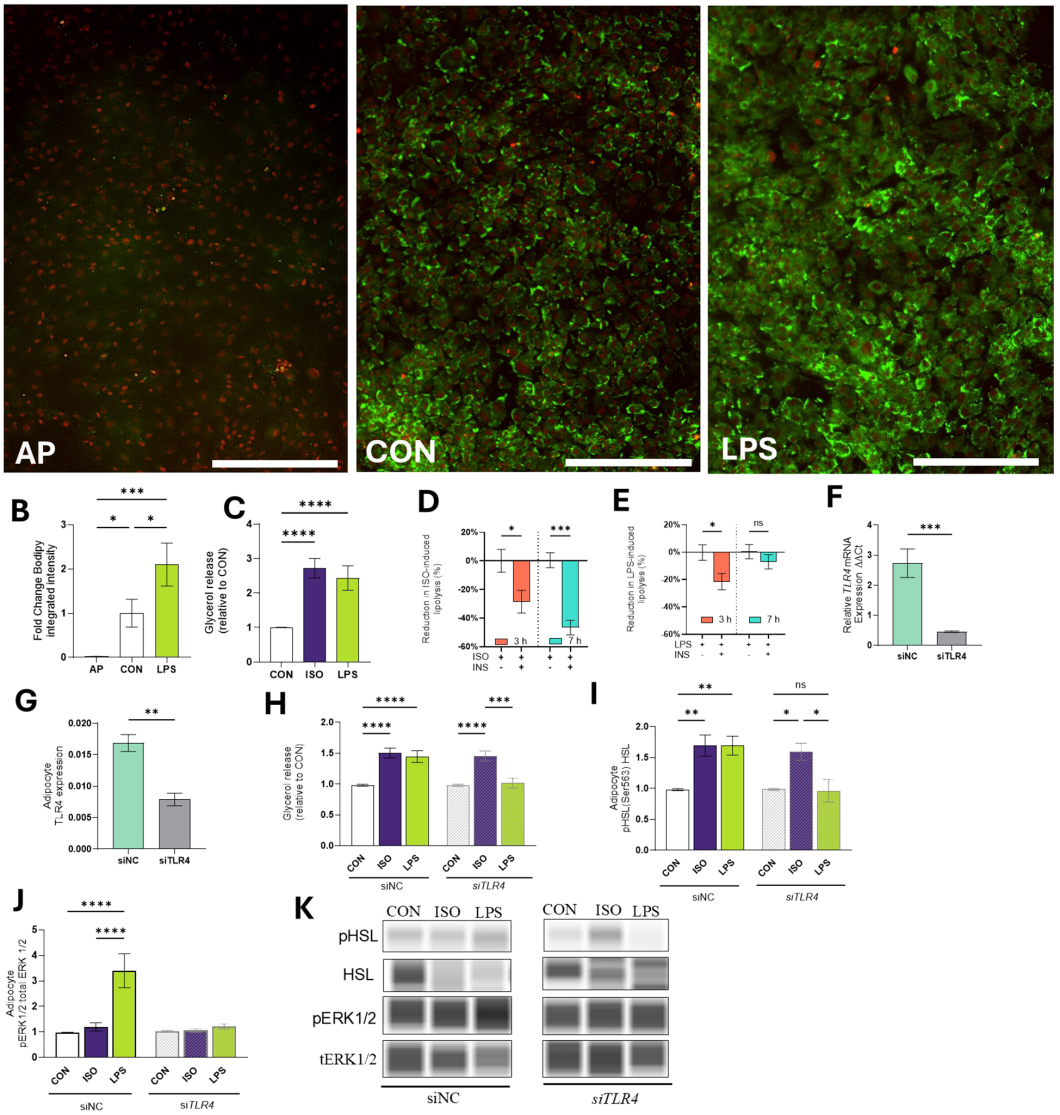
Effects of LPS on lipid mobilization in bovine adipocytes. **(A)** Representative images of bovine adipocyte progenitors (AP) and differentiated adipocytes cultured for 4 d in control (CON) pro-adipogenic media or pro-adipogenic media and LPS (1 µg/mL). Lipid droplets (green) were stained with Bodipy 493/503 and nuclei (red) were stained with NucSpot 650. **(B)** Integrated intensity of bodipy was calculated by the IncuCyte zoom software (Mean intensity x area). **(C)** Glycerol release in cell culture media relative to Control (CON) in differentiated (7-d) bovine adipocytes treated with LPS (1 µg/mL) or isoproterenol (ISO=1µM) for 7 h. Adipocytes were pre-incubated with or without insulin (INS=1 µg/L) for 30 min and treated during 3 and 7 h with ISO or LPS INS-mediated glycerol reduction during ISO-induced lipolysis **(D)** and LPS-induced lipolysis **(E)**. **(F)** Transcription of *TLR4* in preadipocytes treated with non-coding siNC or siTLR4 (siRNA *TLR4*). Gene expression was quantified 7 d after silencing and normalized with reference genes *EIF3K, RPL19*, and *RPS9* using the 2^(- ΔΔCT) method. **(G)** Protein expression of TLR4. **(H)** Glycerol release in 7d differentiated adipocytes treated with non-coding siNC or siTLR4. **(I)** Protein abundance of HSL and phosphorylated HSL (Ser 563) relative to CON. Bands were detected at 81 kDa. **(J)** Protein abundance of extracellular signal-regulated kinases 1/2 (ERK1/2) and phosphorylated ERK1/2 (pERK1/2) at Thr202/tyr204 relative to CON. Protein data are means of the ratio phosphorylated: total protein. Bands were detected at 44 kDa. **(K)** Representative blots obtained in Abby protein Simple System. Error bars represent ± SEM. Bars with * differ (P<0.05), ** (P<0.01), *** (P<0.001), or **** (P<0.0001), n = 6. Scale bar = 400 microns **(A)**.

To evaluate the effect of endotoxin exposure on AP adipogenesis, we induced AP to differentiate into adipocytes for 4 d and quantified lipid accumulation. Compared to undifferentiated AP, CON adipocytes had 47-fold higher lipid accumulation (*p* < 0.05; **Figures 3A, B**). LPS treatment resulted in a 2-fold change in lipid accumulation, compared to CON (*p* < 0.0001; **Figures 3A, B**).

Evaluation of adipocyte lipolytic responses indicated that exposure to LPS for 7h increased lipolysis (measured as glycerol release) at levels comparable to those induced by ISO (p > 0.1; **Figure 3C**). Compared to CON, LPS increased glycerol release 72.66 ± 15.1% (*p* < 0.0001; **Figure 3C**).

Next, we assessed adipocyte responses to insulin by assessing the reduction of lipolytic responses (as determined by glycerol release into the cell media). The inclusion of INS during ISO-induced lipolysis effectively reduced glycerol release at 3 h and 7 h by 20.6 ± 7.9% and 46.5 ± 5.2%, respectively (*p* < 0.05; **Figure 3D**). INS reduced LPS-induced lipolysis at 3 h by 21.5 ± 7.9 (*p* < 0.05). However, INS did not inhibit lipolysis when cells were exposed to LPS for 7 h (-0.44 ± 5.2%, *p* > 0.1; **Figure 3E**).

#### 3.2.3 LPS activates lipolysis by TLR4 signaling

To investigate the role of TLR4 on lipolysis, AP cells were treated with si*TLR4.* Throughout the 7-d culture period, siTLR4 cells exhibited 82.31% reduction in *TLR4* expression compared to siNC cells (*p* < 0.001; **Figure 3F**). Similarly, the TLR4 protein expression was reduced by 54.2% (p < 0.01; **Figure 3G**). Lipolysis was reduced in siTLR4 cells in the presence of LPS. In siNC cells, lipolysis increased by 1.42- and 1.50-fold when incubated with LPS and ISO, respectively (*p* < 0.0001; **Figure 3H**). In line with this, LPS and ISO enhanced HSL phosphorylation by 1.66 and 1.64-fold in siNC (*p* < 0.01). In contrast, HSL phosphorylation was inhibited in siTLR4 adipocytes treated with LPS (**Figure 3I**). In the presence of LPS, greater ERK1/2 phosphorylation was observed, however, siTLR4 attenuated ERK1/2 phosphorylation (*p* < 0.0001; **Figures 3J, K**).

#### 3.2.4 LPS mediates the production of PGE_2_ in adipocytes

**Figure 4:**
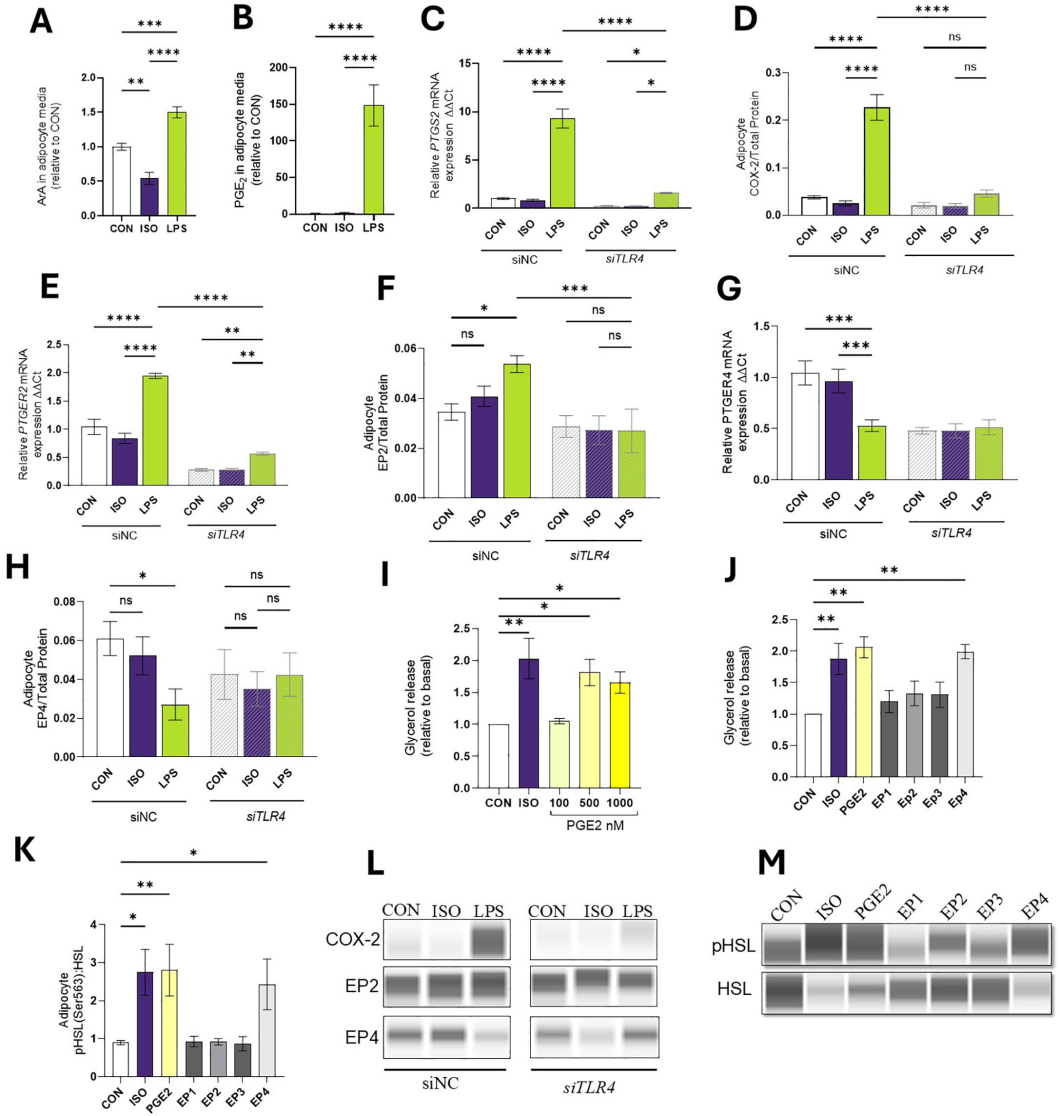
LPS Mediates the Production of COX-2 derived products in Bovine Adipocytes. Differentiated (7-d) bovine adipocytes (n = 6) treated with media control (CON), Lipopolysaccharide (LPS = 1 µg/mL) or isoproterenol (ISO = 1 µM) for 7 h. **(A)** Achidonic acid [ArA, C20:4 (n-6)] in cell culture media (ng/mL). **(B)** Prostaglandin E2-PGE2 (ng/mL). Values are least squares means concentrations in adipocyte culture media measured in HPLC MS/MS) and normalized with CON. Differentiated (7-d) bovine adipocyte media) treated with non-coding small interference RNA (siNC) or targeting *TLR4* (siTLR4) were incubated with media CON, LPS, or ISO for 7 h. **(C)** mRNA expression of prostaglandin-endoperoxide synthase 2 (*PTGS2) a.k.a cyclooxygenase 2 (COX-2)*, **(D)** protein expression COX-2 **(E)** mRNA expression of prostaglandin receptor E2 (*PTGER2)*, **(F)** EP2 protein expression **(G)** mRNA expression of prostaglandin receptor E4 (*PTGER4)*, **(H)** EP4 protein expression **(I)** Glycerol release in 7d differentiated adipocytes treated PGE2. **(J)** Glycerol release in 7d differentiated adipocytes treated with ISO, PGE2 (500 nM), and 10 mM of EP agonists 17-phenyl trinor Prostaglandin E2 (EP1), 11-deoxy Prostaglandin E1 (EP2), Sulprostone (EP3), and CAY10684 (EP4). **(K)** Protein abundance of HSL and phosphorylated HSL (Ser 563) relative to CON. **(L, M)** Representative blots obtained in Abby protein Simple System. Gene expression relative to CON 2^(- ΔΔCT). Gene expression was normalized by reference genes *EIF3K, RPL19*, and *RPS9*. Error bars represent ± SEM. Bars with * differ (P<0.05), ** (P<0.01), *** (P<0.001), or **** (P<0.0001), n = 6.

We analyzed the abundance of specific FA and oxylipins in cell culture media. Adipocytes exposed to LPS released 1.5 ± 0.27 more arachidonic acid [ArA, C20:4, n-6] in the media than CON (*p* < 0.01; **Figure 4A**). Compared to CON, LPS treatment increased PGE2 production by 148.4-fold (*p* < 0.0001; **Figure 4B**).

#### 3.2.5 LPS modulates COX-2 and prostaglandin receptors 2–4 expression in bovine adipocytes in a TLR4-dependent manner

In line with PGE_2_ production, adipocytes exposed to LPS demonstrated enhanced transcription and translation of *PTGS2* and COX-2 respectively (*p* < 0.0001). siTLR4 cells exhibited a 73.2% reduction in *PTGS2* expression and a 46% reduction in protein expression when exposed to LPS (p < 0.0001; **Figures 4C, D**). In line with the RNAseq results, LPS induced upregulation of *PTGER2* gene (p < 0.0001) and COX-2 protein expression in siNC cells, but in si*TLR4*, *PTGER2* expression was reduced under CON, ISO, and LPS conditions (p < 0.05; **Figures 4E, F**). In siNC cells, LPS reduced *PTGER4* transcription (p < 0.0001). However, in si*TLR4*, attenuation of *PTGER4* expression was observed across all treatment groups, and LPS failed to reduce *PTGER4* levels below CON or ISO. At the protein level, a similar pattern of *PTGER4* expression was observed (p < 0.05; **Figures 4G, H, L**).

#### 3.2.6 PGE_2_ activates lipolysis

To understand the role of PGE_2_ on lipid mobilization, mature adipocytes were exposed to different concentrations of PGE2 (**Figure 4I**). Compared to CON, PGE_2_ (500 nM) increased lipolysis by 80.3% (p < 0.05). Adipocytes pre-treated with EP agonists were used to quantify the contribution of the receptors on lipolysis. Compared to CON, only EP4 agonist CAY10684 activates lipolysis by 99% and enhances the pHSL: HSL ratio (p < 0.01; **Figures 4J, K, M**).

#### 3.2.7 COX inhibition reduces LPS-induced lipolysis

**Figure 5:**
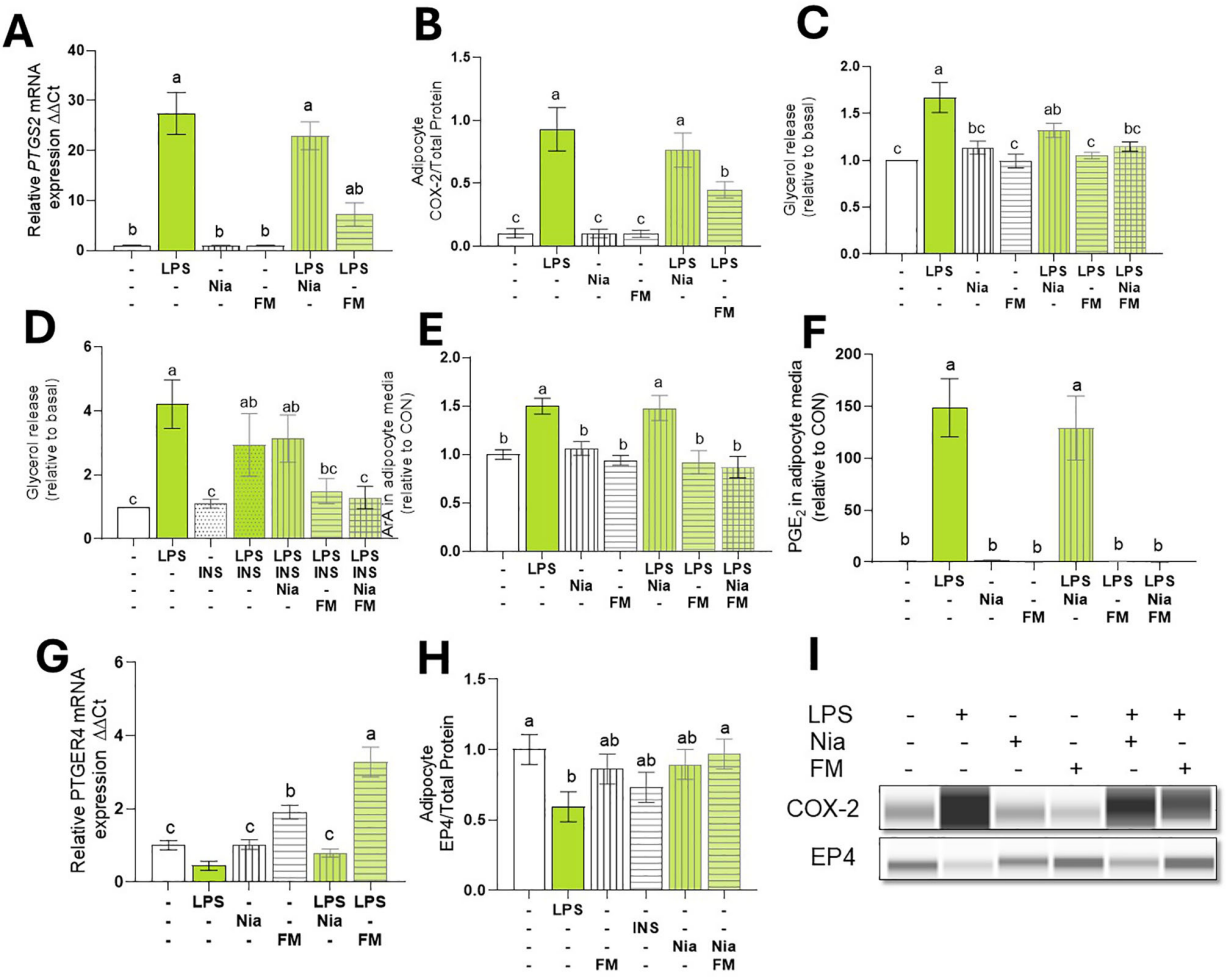
COX inhibition Reduced LPS-induced lipolysis and PGE2. Differentiated (7-d) bovine adipocyte media (n = 6) were preincubated incubated with niacin (NIA: 100 µM), flunixin meglumine (FM: 10 µM), or Insulin (INS: µg/L) for 2 h. Lipolysis was induced with lipopolysaccharide (LPS = 1 µg/mL) for 7 h. **(A)** mRNA abundance of prostaglandin-Endoperoxide Synthase 2 (*PTGS2) a.k.a cyclooxygenase 2 (COX2).* **(B)** COX-2 protein expression. **(C, D)** Glycerol release. **(E)** *ArA:* arachidonic C20:4 in adipocyte culture media. **(F)** PGE2: Prostaglandin E2 in adipocyte culture media. **(G)** mRNA abundance of prostaglandin receptor E4 (*PTGER4).* **(H)** EP4 Protein expression. **(I)** Representative blots obtained in Abby protein Simple System. *G*ene expression. was normalized by reference genes *EIF3K, RPL19*, and *RPS9* and expressed relative to CON (2- ΔΔCT). Error bars represent ± SEM. Bars with different letters differ significantly (a,b,c *P* < 0.05) n = 6.

Treating mature adipocytes with the COX inhibitor FM reduced *PTGS2* transcription and COX-2 abundance. This reduction was observed even in the presence of LPS (p < 0.001; **Figures 5A, B**). As expected, the lipolysis inhibitor NIA did not alter LPS-induced *PTGS2* transcription (p > 0.1).

To assess the role of COX activation on lipolysis, bovine adipocytes were pretreated with FM and NIA for 2 h. As expected, NIA reduced ISO-induced lipolysis to baseline levels. Likewise, the NIA+FM combination suppressed ISO-induced lipolysis (data not shown). However, in the presence of LPS, NIA did not inhibit lipolysis and, instead, was 31% higher than CON. In contrast, FM reduced LPS-induced lipolysis to levels similar to CON (p < 0.001; **Figure 5C**).

To examine the impact of NIA and FM on adipocyte insulin sensitivity during inflammation, we incubated the cells with NIA, FM, or both for 2 h. After treatment with INS, lipolysis was stimulated using LPS. In adipocytes pretreated with NIA, INS did not decrease lipolysis. However, adipocytes treated with FM exhibited responsiveness to the antilipolytic effect of insulin (p < 0.05; **Figure 5D**).

#### 3.2.8 COX inhibition blocks LPS-induced ArA oxylipin production

To evaluate the impact of COX inhibition on prostanoid production in adipocytes, cells were treated with FM, and levels of ArA and PGE2 were quantified. During LPS stimulation, FM reduced adipocytes’ release of ArA and PGE_2_ (p < 0.005; **Figures 5E, F**). Furthermore, FM independently enhanced the expression of PTGR4 and effectively reversed the LPS-induced downregulation of PTGR4 (p < 0.0001; **Figures 5G, H, I**).

## 4 Discussion

### 4.1 Endotoxemia induces WAT inflammation

Data from the present study demonstrate LPS-induced endotoxemia has significant effects on systemic and local (WAT) inflammation and metabolism in dairy cows, paralleling findings in humans and other monogastric species. The rapid increase in rectal temperature, respiration rate, and circulating acute phase proteins (LBP, SAA, Hp) observed in our bovine model replicates the rapid and robust immune responses previously reported in other models of endotoxemia (8, 37).

These results also replicate the shift in energy partitioning that prioritizes glucose towards immune function and away from milk production, as reflected in the reduction in milk production following LPS challenge —a response that is expected after periods of anorexia triggered by immune activation (38). Increased NEFA levels further indicate that lipolysis is enhanced during inflammation. These observations align with previous studies in humans, which report increased serum NEFA 4 hours after IV endotoxin administration, and in rats, 12 hours after administration (39, 40). In addition, we recently demonstrated in the same group of cows used herein that LPS increases the concentration of fatty acids in plasma 2 h post-infusion—including arachidonic (C20:4, n-6; ArA), α-linolenic (C18:3, n-3; ALA), docosahexaenoic (C22:6, n-3; DHA), docosapentaenoic (C22:5, n-3; DPA), eicosapentaenoic (C20:5, n-3; EPA), and linoleic (C18:2, n-6; LA). These findings highlight that endotoxemia may induce an early increase in lipolytic responses, rapidly altering systemic fatty acid profiles (Myers et al. *in review*).

The increase in macrophage infiltration in WAT after 24 h of LPS infusion in the present study recapitulates the inflammatory response and the accumulation of macrophages within WAT during endotoxemia reported in humans (8, 41). It is known that macrophage trafficking into WAT during endotoxemia is triggered by the activation of CD14 receptors, which contributes to a pro-inflammatory environment within WAT, exacerbating insulin resistance and metabolic dysfunction during endotoxemia (41). Notably, humans can exhibit WAT inflammation as early as 4 h after endotoxemia (8).

Data from the transcriptomic analysis of adipocyte responses to LPS presented in this study align with the inflammatory response observed in monogastric WAT (8, 42, 43), which demonstrate that LPS exposure upregulates immune-related gene transcription across species. Notably, the involvement of insulin resistance pathways highlights the potential for endotoxin exposure to disrupt adipocyte metabolic function, which has broader implications for systemic metabolic health during inflammatory conditions. Moreover, the well-documented alterations in insulin signaling during inflammation further validate the use of bovine adipocytes as a model to study WAT dysfunction.

### 4.2 Dual effects of endotoxin on lipolysis: early activation and later suppression

The present study characterized for the first time the lipolytic response and the underlying mechanism of LPS-induced lipolysis in bovine adipocytes. Additionally, this study provides evidence for an initial lipolytic response to LPS that is subsequently reduced over time. Similar to rodents, LPS doses in the µg/mL range trigger lipolysis. For instance, a dose as low as 0.1 µg/mL of LPS activated lipolysis in human adipocytes over a 24 h period (40). Interestingly, human adipocytes appear to be more sensitive to the lipolytic effect of LPS, as 0.01 µg/mL of LPS triggers a robust lipolytic response over a 24-h period (44). The lipolytic responses induced by LPS-mediated TLR4 activation observed in the current study are comparable to those produced by β-adrenergic stimulation. Importantly, TLR4 serves as the primary receptor for gram-negative bacterial endotoxins and plays a central role in immune signaling across many cell types, including adipocytes (45). In the present study in si*TLR4* adipocytes, LPS failed to induce lipolysis and activation of HSL and ERK1/2. However, si*TLR4* adipocytes exhibited a normal lipolytic response when exposed to ISO. Therefore, TLR4 activation appears to be a rate-limiting factor in LPS-mediated lipolysis. Notably, our results align with previous observations in TLR4 mutant mice that exhibited reductions in lipolysis both *in vivo* and *in vitro* after endotoxin challenge, suggesting this response is conserved across species (40).

Upstream regulation of lipolysis occurs by canonical and inflammatory pathways that converge at the phosphorylation of HSL (44). Therefore, the final proxy to quantify lipolysis activation is through HSL phosphorylation the main neutral lipase responsible for the hydrolysis of triglyceride molecules (46). The attenuation of glycerol release and reduced ERK1/2 and HSL phosphorylation observed herein align with previous reports, emphasizing that LPS-induced lipolysis is ERK1/2 dependent (40, 47). We observed that LPS administration suppressed ex vivo basal (CON) and canonical stimulated lipolysis (ISO) in WAT 24 h after infusion, likely due to the lower ATGL content and limited phosphorylation of HSL (i.e., pHSL) (14). The observed reduction in canonical lipolysis following endotoxemia is suggestive of the development of catecholamine resistance. In humans, catecholamine resistance results from increased PDE3B activity driven by prolonged activation of the NFκB pathway, which hydrolyzes cAMP during inflammation (48). Importantly, reduced lipolysis may contribute to altered adipocyte and WAT functionality. Adipocyte size, often used as an indirect measure of TG accumulation, is a key indicator of metabolic disruption. Although significant changes in adipocyte size were not observed following LPS administration, this could be attributed to the small sample size and the short duration of our study, limiting our ability to detect subtle differences. In contrast, prolonged (4-wk), continuous LPS infusions were previously shown to increase the abundance of small adipocytes in mice, suggesting that, long-term, endotoxemia may lead to pronounced alterations in adipocyte morphology and lipid storage (49).

To our knowledge, this is the first study to assess the effects of endotoxemia on lipase activity beyond 16 h post-endotoxemic challenge. Previous studies in humans have demonstrated that HSL activation increases in WAT 2.5 h after a bolus administration of LPS (39). Similarly, a study in mice demonstrated that endotoxemia increases HSL activity within the first 4 h, followed by a decline at 16 h, whereas ATGL activity remained elevated up to 16 h after endotoxin infusion (50). Our data suggest a shift in lipase activity occurs during endotoxemia, transitioning from heightened lipolysis during the early hours to a transient reduction in both basal and canonical lipolysis later on. Reductions in lipase activity during inflammation have previously been linked to a regulatory mechanism that limits lipolysis and the availability of ArA-derived products in mast cells, helping to resolve inflammation (19). Thus, our findings in cows suggest a similar mechanism may govern the inflammatory response in WAT and reduce lipolysis in adipocytes.

### 4.3 Insulin signaling during endotoxin-induced inflammation

Insulin inhibits lipolysis by activating Akt and PDE3B, suppressing HSL activity (51). During the postpartum period, cows are less sensitive to insulin as part of a homeorhetic adaptation that allows lipolysis from WAT to fulfill energy requirements (52). However, insulin resistance can have deleterious effects as it promotes excessive and protracted lipolysis, increasing the risk for metabolic diseases. In the present study, insulin suppressed canonical lipolysis; however, LPS exposure impaired insulin’s ability to inhibit lipolysis, indicating the development of insulin resistance. These findings also align with our group’s previous observations in WAT explants exposed to LPS (18). Although the direct mechanisms of insulin resistance were not investigated in the present study, extensive evidence suggests that, during LPS exposure, increased production of proinflammatory cytokines including TNF-α and IL6 are responsible for downregulation of PDE3B, hindering lipolysis inhibition (51). Similarly, inflammatory pathways such as TNF, JNK, and suppressor of cytokine signaling (SOCS), which are activated by LPS, aberrantly phosphorylate IRS, leading to reduced insulin signaling and the development of insulin resistance (53).

### 4.4 Endotoxin alters COX-prostaglandin E2 pathway

In line with previous studies, our results indicate that COX-2 activation is dependent on TLR4 activation (54). This rapid induction is mediated through key inflammatory pathways, including MAPK and NF-κB signaling, which are activated by the binding of LPS to TLR4 (55, 56). In line with this, our results provide evidence that COX-2 activation is also dependent upon TLR4 activation, as lower COX-2 expression was observed in *siTLR4* cells. As expected, the abundance of COX-2 is greater in TLR4-expressing adipocytes exposed to LPS, which resulted in higher systemic concentrations of its lipid mediator derivatives, including PGE_2_, PGD_2_, and 6-keto-PGF1α (Myers et al. *in review*). These results are consistent with the well-established role of COX-2 in facilitating the rapid production of PG, which contributes to the overall inflammatory response in WAT (57). Beyond the role of lipases such as HSL and ATGL, the observed higher transcription of phospholipases A2, suggests that these enzymes also contribute to the release of ArA from membrane phospholipids, providing an additional source for PG synthesis. However, the observed downregulation of COX-2 in WAT following 24 h of LPS administration suggests a shift in COX-2 signaling dynamics during endotoxemia. This response may represent a negative feedback mechanism to attenuate excessive inflammation and lipolysis. In human macrophages, *PTGS2* mRNA and COX-2 protein levels rise as early as 2 h post-LPS exposure, peak at 6 h, and decline by 24 h, indicating comparable kinetics across different cell types (58). The variations in COX-2 abundance may also account for the differing observations in early activation and later suppression of lipolysis, as PGE_2_ may activate lipolysis (59). In fact, COX-2 deficiency blocks lipolysis while its overexpression reduces adipose mass in a PGE_2_-dependent manner (60, 61). In line with this notion, we observed enhanced lipolysis in cells treated with PGE_2_ and an EP4 receptor agonist.

The observed downregulation of EP4 in the presence of LPS suggests that WAT response to the effects of PGE_2_ may be limited during the latter stages of inflammation. This finding may hold important implications for lipid metabolism, as PGE_2_ signaling through EP4 enhanced adipocyte lipolysis in the present study. The observed reduction of EP4 expression in both WAT and adipocytes requires further investigation as it may be a key driver in the reduction of lipolysis. Together, these findings highlight a novel mechanism by which endotoxins and inflammation reduce lipolysis and enhance lipid accumulation by affecting EP4 abundance. To our knowledge, this is the first study to demonstrate the capacity of endotoxemia to reduce mRNA and protein expression of EP4 in WAT and bovine adipocytes. In microglial cells, LPS-induced inflammation upregulates EP2 expression and downregulates that of EP4 (62). This suggests a potential mechanism by which inflammation may regulate lipolysis by altering EP abundance in WAT.

In summary, our data suggest that TLR4 activation initiates a signaling cascade involving COX-2-dependent PGE2 synthesis, activating EP receptors to regulate lipolysis. Specifically, EP4 receptor activation appears necessary for the initial lipolytic response, potentially through the activation of protein kinase A (PKA) and subsequent phosphorylation of hormone-sensitive lipase (HSL). However, prolonged LPS exposure downregulates EP4 expression while maintaining EP2, shifting the balance toward lipid accumulation. This shift may involve alternative signaling pathways, such as phospholipase C (PLC) and protein kinase C (PKC), which have been implicated in adipocyte lipid storage. Further research is warranted to delineate the intracellular networks mediating this biphasic response.

Flunixin meglumine is a potent anti-inflammatory agent commonly used in cattle. It inhibits COX enzymes, reducing the production of PG. As demonstrated in the present study; by inhibiting COX activity, FM effectively blocks the stimulation of lipolysis by inflammatory products including PGE_2_ (59). These results further emphasize that LPS induces lipolysis through the inflammatory pathway rather than the canonical, PKA-dependent pathway. To the best of our knowledge, this is the first study to investigate the effect of COX inhibition on inflammatory lipolysis in adipocytes. A recent study investigating the selective inhibition of COX-2 activity with celecoxib in 3T3-L1 adipocytes revealed that COX-2 inhibition does not affect ISO-induced canonical lipolysis (21). Likewise, our findings support this observation, as we also observed that FM did not inhibit ISO-induced lipolysis. Although conjecture, these observations could be explained by the potent inhibitory activity of FM on COX enzymes, which lowers PGE_2_ release from adipocytes.

Niacin is a potent antilipolytic agent approved for use in lactating dairy cows. It inhibits the canonical lipolytic pathway upon binding the G protein-coupled receptor 109A (GPR109A), limiting PKA activation in adipocytes (63). As expected, we observed that while NIA inhibited canonical lipolysis, it did not exhibit the same inhibitory effect on LPS-induced lipolysis in adipocytes. This finding suggests that LPS-induced lipolysis occurs independently of the PKA-HSL axis.

### 4.5 Physiological relevance

In this study, we evaluated the effects of LPS-induced endotoxemia on WAT lipolysis and PG signaling in a bovine model. Our findings demonstrate that endotoxemia profoundly disrupts WAT metabolism by affecting both basal and stimulated lipolysis. Notably, our results highlight the critical role of the PGE2 pathway and TLR4 signaling in the activation of lipolysis. Following the acute inflammatory phase, endotoxemia reduces lipolysis intensity and alterations in EP receptor activity.

These changes may help attenuate inflammation by limiting the release of key fatty acids, including PUFA. Additionally, our results indicate that endotoxemia also triggers the activation of lipogenesis. Given the diverse functions of PGE2, dysregulation of EP receptors could further impact WAT function, influencing lipid metabolism and inflammatory signaling pathways. The physiological consequences of reduced lipolysis during endotoxemia extend beyond adipose tissue metabolism. A decrease in lipid mobilization could impair the availability of fatty acids as an energy source, exacerbating negative energy balance, particularly in lactating dairy cows that rely on efficient lipid utilization. Moreover, impaired lipolysis may influence immune function by altering the availability of lipid-derived mediators that regulate and resolve inflammation. This shift in lipid metabolism may also contribute to lipid accumulation and insulin resistance, conditions commonly observed in mammals with chronic inflammatory status (64).

From a therapeutic standpoint, targeting prostaglandin receptors to modulate lipid mobilization presents opportunities and challenges. While selective EP receptor agonists or antagonists could help fine-tune lipolysis, their specificity and systemic effects need careful consideration. For instance, EP receptors are involved in various physiological processes, including vasodilation, immune modulation, and gastrointestinal function, raising concerns about potential side effects. An alternative approach could involve dietary strategies or pharmacological modulation of COX-2 activity to regulate PGE2 synthesis more broadly. Future research should explore these therapeutic avenues to mitigate the metabolic consequences of endotoxemia in dairy cows and potentially other species.

## Data Availability

The datasets presented in this study can be found in online repositories. The names of the repository/repositories and accession number(s) can be found below: https://www.ncbi.nlm.nih.gov/geo/, GSE267141.

## Supplementary material

Supplementary Figure 1 Effects of IV infusion [lipopolysaccharide (LPS) or Saline solution (SS)] on **(A)** Respiratory rate, **(B)** feed intake (DMI), **(C)** Milk yield, **(D)** Glucose, **(E)** Total calcium, **(F)** Magnesium. Time P1 represents the average of measurements obtained during period 1 and was not used as a covariate. Data are represented as mean ± SEM and considered significant if * differ *P* < 0.05, ** *P* < 0.01, or *** (*P* < 0.001), n = 4.
