# Supplementary figures and images for: TLR4 and prostaglandin pathways at the crossroads of endotoxemia-induced lipolysis
Source: Front Immunol. 2025 May 19;16:1591210. doi: 10.3389/fimmu.2025.1591210 (PMC12127734; doi:10.3389/fimmu.2025.1591210)

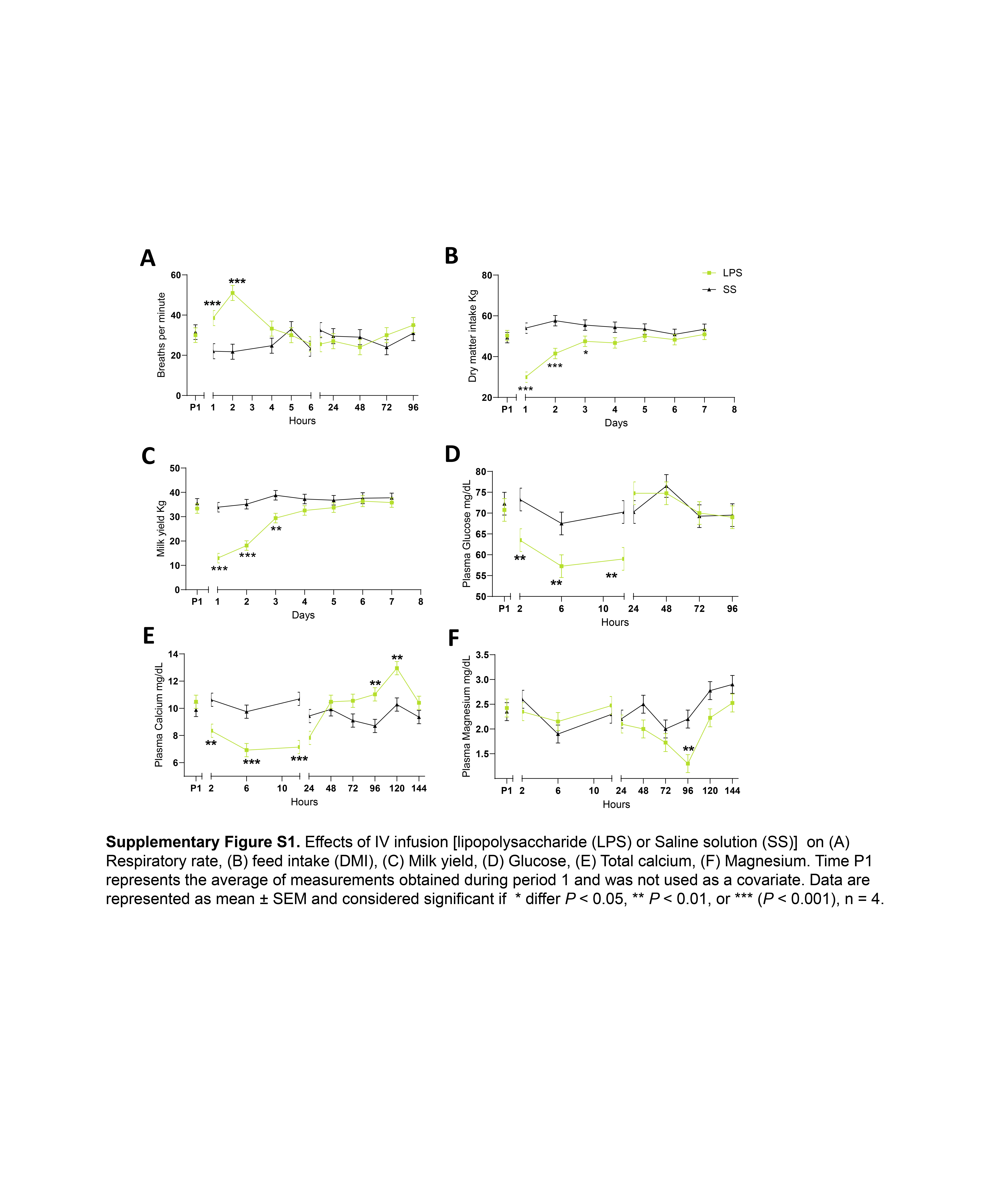

Supplement: Supplementary Figure 1 — Effects of IV infusion [lipopolysaccharide (LPS) or Saline solution (SS)] on (A) Respiratory rate, (B) feed intake (DMI), (C) Milk yield, (D) Glucose, (E) Total calcium, (F) Magnesium. Time P1 represents the average of measurements obtained during period 1 and was not used as a covariate. Data are represented as mean ± SEM and considered significant if * differ P < 0.05, ** P < 0.01, or *** (P < 0.001), n = 4. [file Image1.jpeg]
