# Supplementary material for: TLR4 and prostaglandin pathways at the crossroads of endotoxemia-induced lipolysis
Source: Front Immunol. 2025 May 19;16:1591210. doi: 10.3389/fimmu.2025.1591210 (PMC12127734; doi:10.3389/fimmu.2025.1591210)
Supplement: Supplementary file 2 [file Table1.docx]

**Supplementary Table S1: Primer sequences**

| Design ID | Ref Seq |  | Sequence 5' to 3' |
| --- | --- | --- | --- |
| *IRS1* | NM_174600.2 | Forward | GACCTCAGCAAATCCTCCTTTA |
|  |  | Probe | ACCAGGACCTCACATCTTCCTCCT |
|  |  | Reverse | GGTGCATCGTACCATCTACTG |
| *PTGER2* | NM_001046300.1 | Forward | CATCACCTTCGCCGTCTG |
|  |  | Probe | AAGTGGGACCTCCAAGCTCTTAGA |
|  |  | Reverse | AGGATGGCAAAGACCCAAG |
| *PTGER4* | NM_001046301.1 | Forward | CGCTTAGGACTCTGCGAATATC |
|  |  | Probe | TTCTTACTGGTGGACGAGGTTGGC |
|  |  | Reverse | TCACTGGGAAACGTGACTTG |
| *PTGS2* | NM_174445.2 | Forward | TGATGACTGCCCAACACC |
|  |  | Probe | TGGGTGTGAAAGGGAGGAAAGAGC |
|  |  | Reverse | CTGGGCAAAGAATGCAAACA |
| *EIF3K* | NM_001034489.2 | Forward | GATCGACTTTGACAGTGTG |
|  |  | Reverse | GCCAAGAAGTCAACACATC |
| *RPS9* | NM_001101152.2 | Forward | GTCTGTTCGAAGGTAATGC |
|  |  | Reverse | CAGGATGTAATCCAGCTTC |
| *TLR4* | NM_174198.5 | Forward | CACTCGCTCCGGATCCTAGA |
|  |  | Reverse | CAAACACAAGCAAATGCATTCTG |
| si*TLR4*  CD.Ri.383506.13.1 |  | Forward | GAGCUUCAAUGAUGUCAUUACCUTA |
|  |  | Reverse | UAAGGUAAUGACAUCAUUGAAGCUCAG |
| si*TLR4*  CD.Ri.383506.13.2 |  | Forward | UGAGCUUCAAUGAUGUCAUUACCT |
|  |  | Reverse | AAGGUAAUGACAUCAUUGAAGCUCAGA |
| si*TLR4*  CD.Ri.383506.13.4 |  | Forward | CUGAGCUUCAAUGAUGUCAUUACT |
|  |  | Reverse | AGGUAAUGACAUCAUUGAAGCUCAGAU |

| Gene | Product | RefSeq |
| --- | --- | --- |
| *EIF3K* | Bt03226565_m1 | NM_001034489.2 |
|  |  |  |
| *RPS9* | Bt03272016_m1 | NM_001101152.2 |
|  |  |  |

**Supplementary Table S2:** Antibody dilutions

| **Antibody** | **Dilution** | **Company** | **Reference/Identifier** |
| --- | --- | --- | --- |
| ATGL | 1:25 | Cell Signaling | #2138 |
| COX-2 | 1:50 | Cell Signaling, Danvers, MA, USA | #12282 |
| Prostaglandin E2 receptor EP2-PTGER2 | 1:50 | Novus Biologicals, Centennial, CO, USA | #SR1927 |
| Prostaglandin E2 receptor EP4-PTGER4 | 1:50 | RayBiotech Life, Inc., Peachtree Corners, GA, USA | #102-14793 |
| HSL | 1:25 | Cell Signaling | #4107 |
| Phosphorylated HSL serine 563 | 1:25 | Thermo Fisher | #PA5-17488 |
| ERK1/1 p44/42-MAPK3/1(Erk1/2) | 1:1000 | Cell Signaling | #9102S |
| Phosphorylated p44/42-MAPK3/1(p-Erk1/2) (T202/Y204) | 1:1000 | Cell Signaling | #9101S |

**Supplementary methods:** Targeted fatty acid lipidomic analysis methods

(as performed by the Lipidomics Core Facility at Wayne State University, Detroit, MI)

Liquid plasma and adipocyte culture medium samples were analyzed directly or after dilution in phosphate buffer (pH 7.2). For fatty acyl lipidomic analysis, samples (1 mL) were spiked with deuterated internal standards, including prostaglandin E1-d4 and arachidonic acid-d8, and polyunsaturated fatty acids and respective metabolites were extracted using C18 solid-phase extraction columns as previously described (1-4). After elution with methanol and drying under nitrogen, residues were reconstituted in methanol-ammonium acetate and subjected to LC-MS/MS analysis. HPLC separation was performed on a Luna C18 column with a gradient mobile phase of methanol, water, and acetonitrile containing ammonium acetate. The eluate was introduced into a QTRAP7500 mass spectrometer in negative ion mode, with conditions optimized for lipid and lipid mediator detection. Multiple Reaction Monitoring (MRM) was used to detect arachidonic acid and prostaglandin E2 based on retention times and characteristic ion transitions matched with the standard (Supplementary table S3). Mass spectra were verified using the Enhanced Product Ion (EPI) feature. The resultant data was processed using Sciex OS 3.4 software, with internal standard signals used for normalization and quantitation.

**Supplementary Table S3:** MRM

| Component name | Internal standard | Expected retention time (min) | Precursor (m/z) | Fragment (m/z) |
| --- | --- | --- | --- | --- |
| PGE2 | PGE1-D4 | 6.43 | 351.2 | 271.1 |
| Arachidonic acid | Arachidonic acid-D8 | 12.50 | 303.2 | 259.2 |

1. Maddipati KR, Zhou SL. Stability and analysis of eicosanoids and docosanoids in tissue culture media. Prostaglandins Other Lipid Mediat. 2011;94(1-2):59-72.

2. Maddipati KR, Romero R, Chaiworapongsa T, Zhou SL, Xu Z, Tarca AL, et al. Eicosanomic profiling reveals dominance of the epoxygenase pathway in human amniotic fluid at term in spontaneous labor. FASEB J. 2014;28(11):4835-46.

3. Maddipati KR, Romero R, Chaiworapongsa T, Chaemsaithong P, Zhou SL, Xu Z, et al. Clinical chorioamnionitis at term: the amniotic fluid fatty acyl lipidome. J Lipid Res. 2016;57(10):1906-16.

4. Maddipati KR, Romero R, Chaiworapongsa T, Chaemsaithong P, Zhou SL, Xu Z, et al. Lipidomic analysis of patients with microbial invasion of the amniotic cavity reveals up-regulation of leukotriene B4. FASEB J. 2016;30(10):3296-307.
